# Evaluations of Acute and Sub-Acute Biological Effects of Narrowband and Moderate-Band High Power Electromagnetic Waves on Cellular Spheroids

**DOI:** 10.1038/s41598-019-51686-9

**Published:** 2019-10-25

**Authors:** Laure Gibot, Jelena Kolosnjaj-Tabi, Elisabeth Bellard, Thomas Chretiennot, Quentin Saurin, Alexandre Catrain, Muriel Golzio, René Vézinet, Marie-Pierre Rols

**Affiliations:** 1 Institut de Pharmacologie et de Biologie Structurale, Université de Toulouse, CNRS, UPS, Toulouse, France; 2 CEA DAM, GRAMAT, F-46500 Gramat, France

**Keywords:** Membrane biophysics, Physics

## Abstract

High power electromagnetic signals can disrupt the functioning of electronic devices. As electromagnetism plays a role in cells homeostasis, such electromagnetic signals could potentially also alter some physiological processes. Herein we report on distinct biological parameters assessment after cellular spheroids exposure to high power electromagnetic signals, such as the ones used for defense applications. Signals effects were assessed in tumor cells spheroids and in normal human dermal fibroblasts spheroids, where macroscopic aspect, growth, plasma membrane integrity, induction of apoptosis, ATP content, and mitochondrial potential were investigated after spheroids exposure to high power electromagnetic signals. No significant effects were observed, indicating that 1.5 GHz narrowband electromagnetic fields with incident amplitude level of 40 kV/m, and 150 MHz moderate-band electric fields with an amplitude of 72.5 to approximately 200 kV/m, do not cause any significant alterations of assessed parameters.

## Introduction

Electronic devices can stop operating in response to intense external electromagnetic signals. Electromagnetic environments, or more precisely intentional electromagnetic environments^1^, are among emerging approaches that could be used for defense applications. High power electromagnetic (HPEM) technology can be deployed to temporarily or permanently disrupt electronic devices or could even be used against the personnel^2^. The HPEM systems that can be used to impair or destroy electronics and hardware^3^, and thus to black out the opponents electronic systems, operate in the range of tens of MHz to a few tens of GHz. Alternatively, HPEM signals (with frequencies of approximately 100 GHz and millimeter waves) could be used against personnel. This non-lethal directed-energy weapon, can heat the surface of its targets (*e.g*. heat people’s skin)^2^. Such non-lethal weapon system, also known as the Active Denial System, was developed by the U.S. military (https://jnlwp.defense.gov/Press-Room/Fact-Sheets/ArticleViewFactsheets/Article/577989/active-denial-technology-fact-sheet/ retrieved August 2018) and was designed for area denial, perimeter security and crowd control. Similar systems were also elaborated by Russia (https://www.popularmechanics.com/military/weapons/a7804/why-russia-will-be-the-first-to-use-the-pain-ray-9833954/ retrieved August 2018) and China (http://en.people.cn/n3/2018/0205/c90000-9423875.html retrieved August 2018).

High Power Microwave (HPM) and High Power Radio Frequency (HPRF) systems can radiate high power and short duration oscillatory pulses of electromagnetic fields. These pulses can exhibit different amplitudes, durations, frequency spectrums, as well as Pulse Repetition Frequencies (PRF), and can be classified into different families, according to their frequency spectrum bandwidth. The bandwidth classification distinguishes the narrowband (also known as hypo band), meso (or moderate) band, ultra-moderate (or sub-hyper) band and hyperband intentional electromagnetic environments^2^.

In parallel, prokaryotic and eukaryotic cells communicate with electromagnetic signals. Electromagnetic fields, generated inside and outside living cells, are implicated in a number of biochemical processes. The interferences with endogenous electromagnetic fields could thus result in modifications of physiological processes, making all living systems susceptible to cues that disturb the electromagnetic homeostasis^4^. As electromagnetism plays a role in the physiology of cells, the presence of external electromagnetic fields could potentially alter physiological processes^4^.

From this perspective, this study aimed to assess the potential biological effects of narrowband (NB) and moderate band (MB) intentional electromagnetic environments. A limited time of exposure (usually lasting only a few seconds) associated with short pulse durations (nanoseconds to a few microseconds), are limiting factors in terms of cumulated radiated electromagnetic energy. In such cases, the tissues do not heat, despite the high amplitude of the radiated electric field (a few hundred of kV/m). Nevertheless, electromagnetic fields generated by external sources, might potentially act on living cells by other, non-thermal mechanisms. As safety is primordial for the protection of civilians and members of the armed services, it is necessary to evaluate the potential biological effects of NB and MB HPEM pulses.

In the present study we evaluated potential biological effects after exposure of healthy and tumor cells spheroids to signals generated by experimental HPEM and HPRF systems in field experiments and on laboratory scale using HPEM applicators^5,6^, which allow reproducing field experiments experimental conditions, in terms of applied pulse shapes and electric field amplitudes.

## Results

### Narrowband and moderate-band illuminations performed in field experiments do not alter spheroids growth

**Figure 1:**
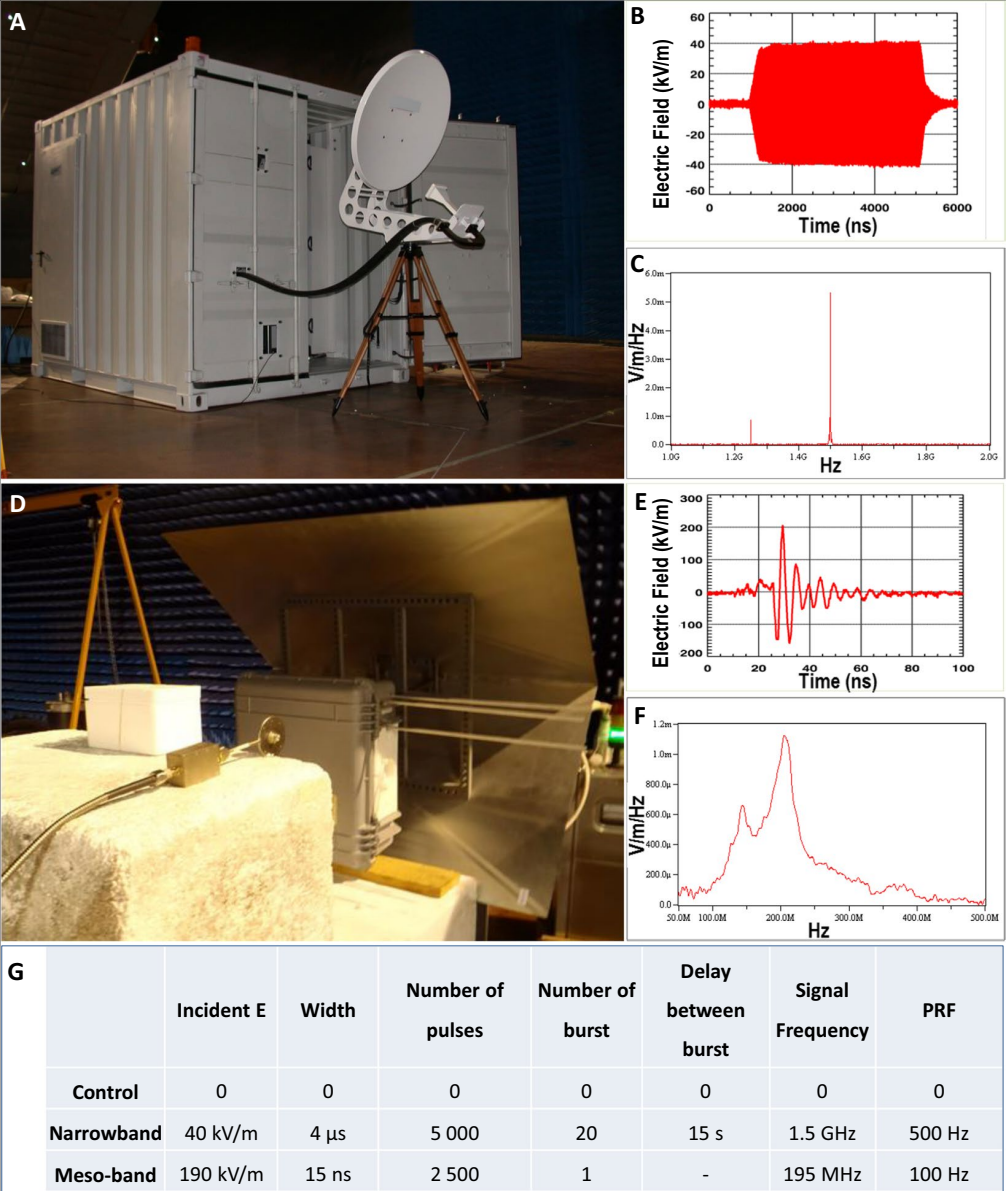
High power systems, antennas and signals applied in field experiments. (**A**) Narrowband system TEMPETE, comprising the parabolic antenna (front) and the High Power system (shelter). (**B**) Electric field radiated by the TEMPETE system. (**C**) Frequency spectrum of the radiated electric field generated by the TEMPETE system. (**D**) Meso-band system DIEHL DS 110 comprising the DIEHL emitter (left) and the DC power supply (right). (**E**) Electric field radiated by the DIEHL DS 110 system. (**F**) Frequency spectrum of the radiated electric field generated by the DIEHL DS 110 system. (**G**) Table summarizing electromagnetic parameters applied in field experiments during narrowband and meso-band illuminations of human normal- and tumor- cell spheroids.

Human cells-derived spheroids were illuminated with narrowband signals generated by the TEMPETE system (Fig. 1A–C) and meso-band signals generated by the DIEHL DS 110 system (Fig. 1D–F). After the application of parameters as specified in Fig. 1G, we monitored spheroids growth over a 10-days period.

**Figure 2:**
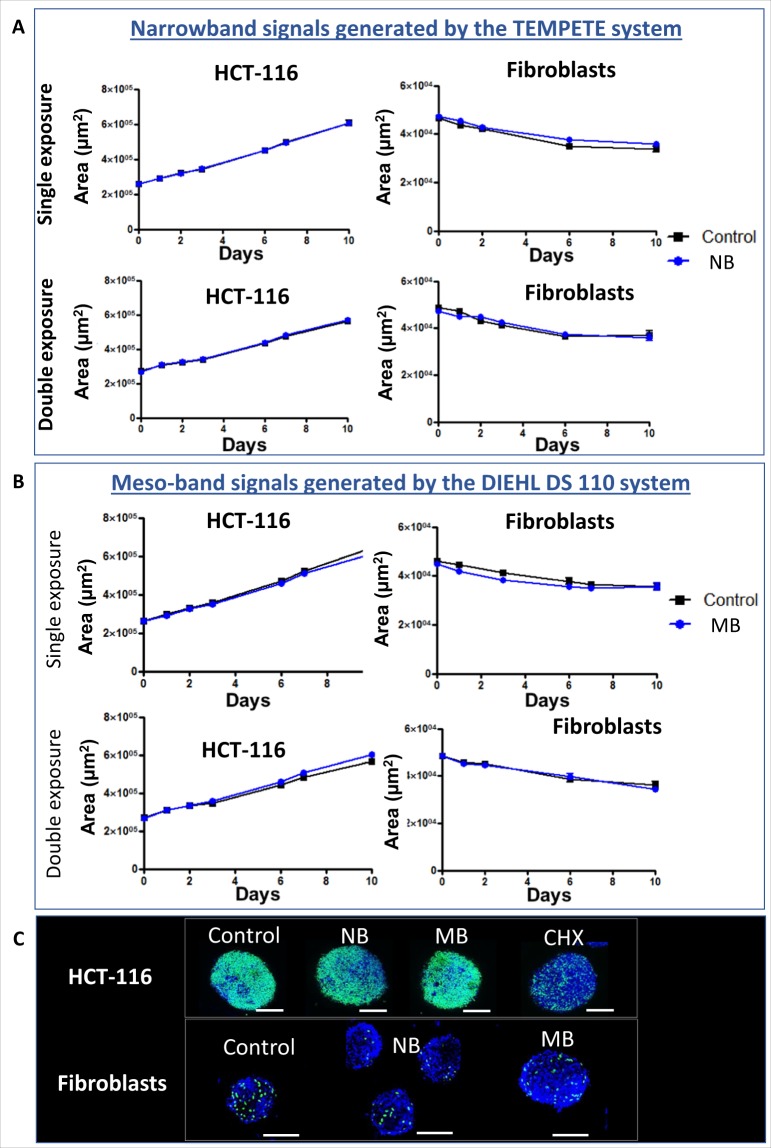
Spheroids growth after exposure to narrowband (NB) and meso-band (MB) illuminations generated in field experiments. Human tumor HCT-116 cells and normal fibroblasts cells spheroids growth after exposure to (**A**) NB signals and (**B**) MB signals. At least six biological replicates were analyzed for each set of experiments. Data are expressed as mean ± SEM and the p-value was set at 0.05. (**C**) Representative fluorescence micrographs of an HCT-116 spheroid (left) and normal dermal fibroblasts spheroid (right) exposed to NB signals, showing the proliferative cells in green color, and the nuclei of non-proliferating cells in a blue color. Scale bar 100 µm.

No effects were observed on spheroids growth, regardless if the spheroids underwent a single illumination protocol or two illumination protocols, performed with a time interval of 24 h (Fig. 2A,B, respectively). Illuminated and non-illuminated cancer or normal cells spheroids exhibited a similar growth pattern. The proliferating cells were located at spheroids’ surface and were more prominent in spheroids containing cancer cells (Fig. 2C). In case of cancer cells spheroids, the curve reflects the increasing proliferation rate of malignant cells. Conversely, in case of fibroblasts spheroids we observed a comparable decrease of spheroids’ growth in non-illuminated and illuminated spheroids. Such growth decline is common in normal cells spheroids, where spheroid growth stops due to contact inhibition^7^.

### Thermal measurements in field experiments

Under our experimental conditions, a temperature increase of 12.6 °C was measured after spheroids exposure to narrowband signals and no temperature increase could be measured after spheroids exposure to meso-band signals.

### Effects of narrowband and moderate-band illuminations performed at the laboratory scale

**Figure 3:**
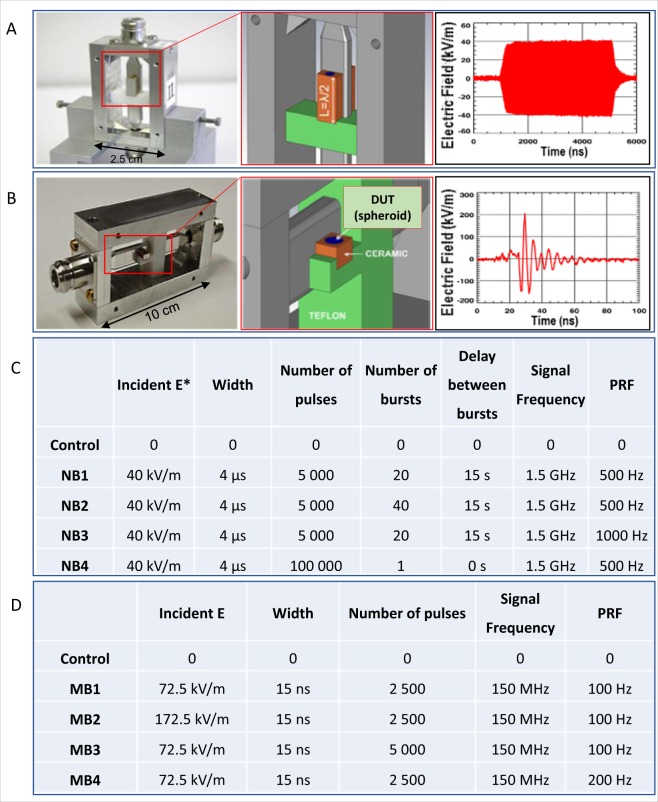
Applicators and signals applied in laboratory experiments. Macroscopic view, schematic detail view and typical signal shape delivered by the applicators for (**A**) 1.5 GHz narrowband signals. The λ/2 length of the ceramic container (brown color in the scheme) constitutes a transmission line impedance transformer at 1.5 GHz (**B**) 150 MHz meso-band band signal. (**A,B**) High permittivity ceramic containers are in contact with the stripline conductors and provide a maximum coupling and homogeneity of the E-field into the biological sample. DUT: Device Under Test. The side covers of the applicators are not present on the images, in order to show the interior, but were mounted whenever the applicators are in use. Tables summarizing the electromagnetic parameters applied in (**C**) narrowband (NB) and (**D**) meso-band (MB) illumination protocols of human normal- and tumor- cell spheroids. (*The Incident E-field value represents the maximum absolute zero to peak value).

Electromagnetic signals, corresponding to electromagnetic waves generated by the the narrowband electromagnetic signals generated by a TEMPETE high power system (GERAC Electromagnetisme, Gramat, France), and meso-band system DIEHL DS 110, were delivered by laboratory compact HPEM pulse applicators (Fig. 3A,B), which were previously presented^5^.

The fields and S parameters calculated with the CST Microwave Studio are shown in supporting information. The parameters, to which cellular spheroids were exposed, are summarized in Fig. 3C,D.

### Narrowband illuminations performed at the laboratory scale do not affect macroscopic aspect, plasma membrane integrity and do not induce apoptosis in normal and tumor human cells-derived spheroids

**Figure 4:**
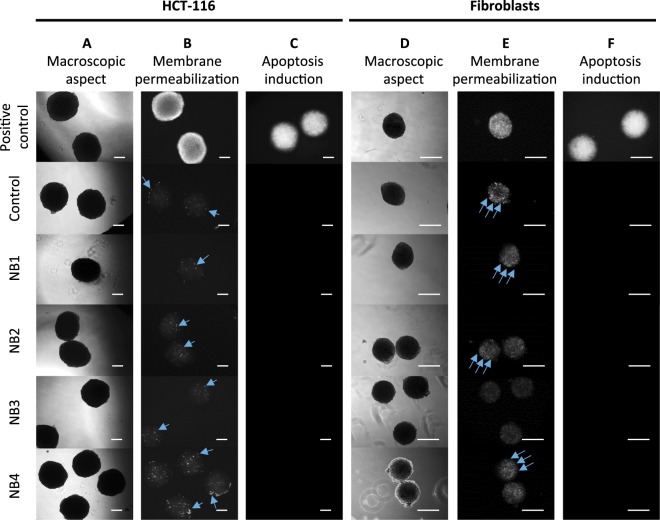
Tumor and normal cells spheroids characterization after narrow band (NB) illumination. (**A–C**) Tumor cell spheroids made with human colorectal cancer cells HCT-116 and (**D–F**) normal human fibroblasts spheroids. (**A,D**) Macroscopic aspect (bright field micrograph) taken 5 minutes after illumination. (**B,E**) Fluorescence micrographs of positive controls submitted to the permeabilizing electric field (1000 V/cm, 100 µs, 8 pulses, 1 Hz), controls and spheroids submitted to NB signal, the images were taken 5 minutes after illumination. (**C,F**) Caspases 3/7 activation 1 h after illumination. Positive controls were incubated with 1 µM Staurosporine for 24 h. Scale bar: 200 µm. Blue arrows indicate some of the fluorescent dots, which correspond to scattered dead cells that internalized propidium iodide. This dead cells occurrence is normal and differs from the global cell permeabilization effect, such as the one observed in positive controls.

Human cells-derived spheroids were illuminated with narrow band signals of fixed electric field strength (40 kV/m), width (4 µs) and signal frequency (1.5 GHz), as well as different numbers of bursts (1 to 40), different number of pulses per burst (from 5000 to 100000) and distinct PRF (500 Hz to 1 kHz), as summarized in Fig. 3C,D. Regardless if human normal cells (fibroblasts isolated from healthy skin) or tumor cells (HCT-116 cell line) spheroids were exposed to HPEM signals, no short-term effect was observed after narrow band illumination in respect to spheroid macroscopic aspect, plasma membrane permeabilization and apoptosis induction (Fig. 4A–F). The shape of HCT-116 and fibroblasts spheroids remained unaltered after illumination. The cells remained bound within the spheroids, and their morphology remained unaltered (Fig. 4A,D). Spheroids that underwent the electropermeabilizing electric field served as positive control. When applied fields were sufficiently strong to permeabilize the membrane, propidium iodide massively entered the permeabilized cells immediately after cells exposure to electric fields, as shown in Fig. 4A,B, top panel. In contrast, after spheroids exposure to NB signals, the membranes of cells in multicellular spheroids did not exhibit a broad permeabilization in comparison to the control (Figure B and E). Here it is worthy to note that dead cell also exhibit plasma membrane defects, leading to some positive staining with propidium iodide. Consequently, some punctual and scattered fluorescent dots were present at the basal level in HCT-116 spheroids (Fig. 4B,E, blue arrows). Similarly, fluorescent signals were observed in all normal fibroblast spheroids, but in illuminated spheroids, the fluorescence signal was not amplified in comparison to controls. In addition to membrane permeabilization effects, the induction of apoptosis was also assessed. The positive control for apoptosis was induced with staurosporine. While high fluorescent signal corresponding to caspases 3/7 activation was observed for this positive control, no signal was visible after narrow band illuminations (Fig. 4C,F). Thus, under mentioned electromagnetic conditions, increasing the number of narrow band pulses or PRF, did not lead neither to membrane permeabilization nor to apoptosis induction.

### Narrowband illuminations performed at the laboratory scale do not alter ATP content, mitochondrial membrane potential and spheroids growth

**Figure 5:**
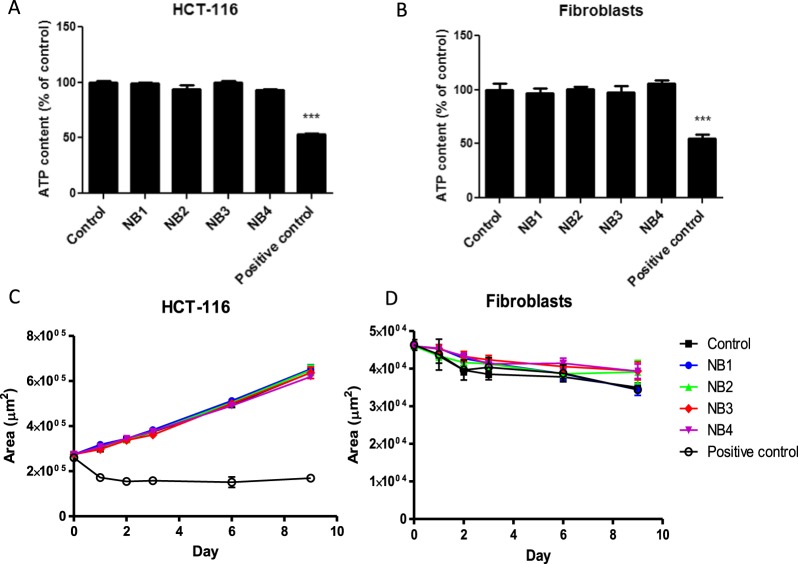
Spheroids ATP content 30 minutes after narrowband (NB) illumination and spheroids growth curves. (**A**) The ATP content in tumor HCT-116 cells spheroids after NB signal illumination. (**B**) The ATP content in normal fibroblasts spheroids after NB illumination. Spheroids submitted to the electric field causing membrane permeabilization were used as positive control. The one-way ANOVA test was followed a Dunnett’s multiple comparison test and the p-value was set at 0.05. (**C**) Tumor HCT-116 spheroids growth curve after NB signals illumination. (**D**) Normal fibroblasts spheroids growth curve after NB illumination. The two-way ANOVA test was performed with a Dunnett’s multiple comparison test. The p-value was set at 0.05 and a significant statistical difference (***P < 0.001) was found between the positive control and all other conditions.

Spheroids ATP content was quantified by a luminescence assay after narrow band signal illuminations. A decrease in ATP content in spheroids could be a sign of ATP leakage through a permeabilized plasma membrane, which potentially could not be revealed by propidium iodide staining, or might be a sign of mitochondrial dysfunction. Under our exposure conditions, no decrease in spheroids ATP content was detected after narrow band illuminations (Fig. 5A,B). Conversely, positive controls, which were electropermeabilized, exhibited ATP leakage. This observation was confirmed by the absence of modification in mitochondrial staining after labeling with a far-red fluorescent mitochondrial dye (the MitoView 633), which is dependent on mitochondrial membrane potential (data not shown).

Finally, the long-term effect of narrow band illuminations on spheroids growth was followed up to 9 days. The spheroids growth curves are represented in Fig. 5C,D. Using a two-way ANOVA statistical analysis coupled to the Dunnett’s test, no statistical difference was observed between the control and narrow band illuminations conditions group. It is worthy to note that while tumor cells spheroids kept growing regularly (Fig. 5C), the spheroids produced with normal cells did not grow in a comparable fashion and slightly decreased in size (Fig. 5D). This is due to the contact inhibition and tissue remodeling processes.

### Meso-band illuminations performed at the laboratory scale do not affect macroscopic aspect, plasma membrane integrity and do not induce apoptosis in normal and tumor human cells-derived spheroids

**Figure 6:**
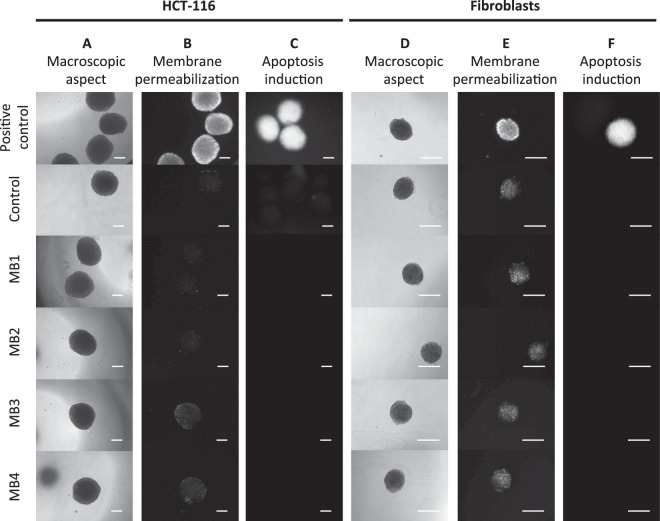
Tumor and normal cells spheroids characterization after meso-band (MB) illumination. (**A–C**) Tumor cell spheroids made with human colorectal cancer cells HCT-116 and (**D–F**) normal human fibroblasts spheroids. (**A,D**) Macroscopic aspect (bright field micrograph) taken 5 minutes after MB illumination. (**B,E**) Fluorescence micrographs of positive controls submitted to the permeabilizing electric field (1000 V/cm, 100 µs, 8 pulses, 1 Hz), controls and spheroids submitted to MB signal, the images were taken 5 minutes after illumination. (**C,F**) Caspases 3/7 activation 1 h after illumination. Positive controls were incubated with 1 µM Staurosporine for 24 h. Scale bar: 200 µm.

Spheroids were illuminated with meso-band signals of fixed signal frequency (150 MHz) and width (15 ns), with the radiated electric field strength ranking from 72.5 kV/m to 172.5 kV/m, different number of pulses (2500 to 5000), and distinct PRF (100 Hz and 200 Hz), as summarized in Fig. 3D. Regardless of the cell type, no significant short-term effect was observed after meso-band illumination in terms of spheroids macroscopic aspect, plasma membrane permeabilization and apoptosis induction (Fig. 6A–F). In both cases (for HCT-116 and fibroblasts-derived spheroids) the shape remained the same before and after illumination, spheroids cells did not detach and the spheroids volume increase was not observed (Fig. 6A,D). Electroporation was applied to spheroids in order to obtain a positive control for plasma membrane permeability investigation. As shown in Fig. 6B,E top panel, propidium iodide massively penetrated into permeabilized cells immediately after electroporation, while insignificant signal was detected after meso-band illuminations, in comparison to the positive controls. The positive control for apoptosis was induced with staurosporine. While high fluorescent signal corresponding to caspases 3/7 activation was observed for this positive control, no signal was visible after meso-band illuminations (Fig. 6C,F). Thus, increasing the field strength or the number of pulses of meso-band signals does lead neither to membrane permeabilization nor apoptosis induction.

### Meso-band illuminations performed at the laboratory scale do not alter ATP content, mitochondrial membrane potential and spheroids growth

**Figure 7:**
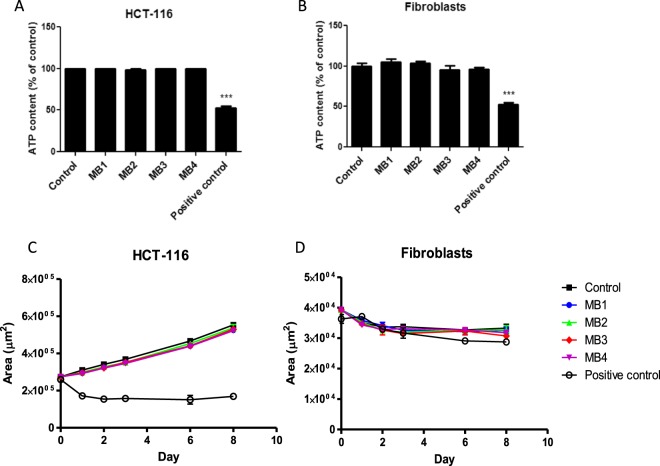
Spheroids ATP content 30 minutes after meso-band (MB) illumination and spheroids growth curves. (**A**) The ATP content in tumor HCT-116 cells spheroids after MB signal illumination. (**B**) The ATP content in normal fibroblasts spheroids after MB illumination. Spheroids submitted to the electric field causing membrane permeabilization were used as positive control. The one-way ANOVA test was followed a Dunnett’s multiple comparison test and the p-value was set at 0.05. (**C**) Tumor HCT-116 spheroids growth curve after MB signals illumination. (**D**) Normal fibroblasts spheroids growth curve after MB illumination. The two-way ANOVA test was performed with a Dunnett’s multiple comparison test and the p-value was set at 0.05.

In the same way as for the narrowband illuminations, no decrease in spheroids ATP content was detected after maso- band illuminations (Fig. 7A,B). Conversely, positive controls, which were electropermeabilized, exhibited ATP leakage. This observation was confirmed by the absence of modification in mitochondrial staining after labeling with a far-red fluorescent mitochondrial dye, which is dependent on mitochondrial membrane potential (data not shown).

In the same way as after narrowband signal exposure, the long-term effect of meso-band illuminations on spheroids growth was followed for 9 days. The spheroids growth curves are represented in Fig. 7C,D. Using a two-way ANOVA statistical analysis coupled to the Dunnett’s test, no statistical difference was observed between the control and meso-band illuminations conditions group.

### Thermal measurements performed at the laboratory scale

The temperature increase after spheroids exposure to meso-band signals was too low to be detected in performed experiments. Conversely, under narrowband signal exposure, the temperature increase of 1.6 °C was measured, after spheroids exposure to narrowband signals (20 bursts of 5000 pulses, delay between bursts 15 s, PRF 500 Hz, 16 kV/m); and a 2.6 °C temperature increase was measured after application of 2 bursts of 50 000 pulses, delay between bursts 15 s, PRF 500 Hz, 20 kV/m. Nevertheless, when the number of pulses was decreased by half and the PRF was decreased to 100 Hz, with the time between pulses 1 s, and E-field strength of 20 kV/m, the temperature increase was smaller than 1 °C.

## Discussion

This study allowed us to assess different exposure levels to HPEM signals in field experiments and at the laboratory scale. At the laboratory scale, different exposure levels (NB1-NB4 and MB1-MB4) were chosen, because they mirror the conditions, which occur in the proximity of TEMPETE and DIEHL HPEM systems. These conditions (40 kV/m peak value for narrow band signals and 175 kV/m peak value for meso-band signals thus correspond to exposures, to which might be exposed the operators of mentioned HPEM systems. Different numbers of pulses, bursts and delays between bursts, which were used in the laboratory tests, reflect the configurations, which might be applied in HPEM signals use real-case scenarios. The performed research allowed us to conclude that 1.5 GHz narrowband signals consisting of pulsed sinusoidal waves with field strengths of 40 kV/m, and 150 MHz meso-band pulsed electric field, consisting of damped modulated sinusoidal waves with field strengths from 72.5 to 172.5 kV/m, do not affect tumor (HCT-116) cells spheroids and normal fibroblasts spheroids morphology and growth. In addition, tested electromagentic signals do not induce the permeabilization of spheroids cells, do not alter the ATP content in spheroids and do not affect the mitochondrial membrane potential. Indeed our study addressed a limited number of parameters, and further studies are required to completely ascertain the biological innocuity of narrowband and moderate band HPEM signals.

Electronic devices are particularly vulnerable to high power electromagnetic radiation waves. Nevertheless, to our knowledge, there is little if any evidence about the effects such radiations could have on humans. Yet, lower frequency electric fields generate current flow throughout living systems, and low-frequency magnetic fields induce circulating currents. Therefore, EMF might potentially affect some biological processes.

Electromagnetic waves (EMWs) act on positively as well as negatively charged compounds, and charged moieties are involved in most biochemical reactions occurring in living systems. Consequently, the EMWs generated by external sources might act on cells, and could be involved in a number of biological processes, which depend on the EMF. These processes include biosynthesis^8^, long-range protein interactions^9^, cell differentiation^10^, neuronal response^11^ and tissue repair^12^, to mention but a few. Indeed, EMFs are stronger in the vicinity of the source, and the field strength rapidly decreases when the distance from the source is increased. Therefore, biological effects could vary with the distance from the EMF emitting point, and induced currents could be very small in respect of the thresholds, which are required to trigger a specific effect.

Furthermore, here we should note that the term “biological effect” should be distinguished from “health effect” or “health risk”. Exposures to EMF in the frequency range from static fields to 300 GHz currently do not allow concluding on potential health risks, according to the opinion of the Scientific Committee on Emerging and Newly Identified Health Risks (SCENIHR) (SCENIHR: Potential health effects of exposure to electromagnetic fields (EMF), 27 January 2015). The International Commission on Non-Ionizing Radiation Protection (ICNIRP) organization also provides this assumption, and states that the overall evaluation of all the research on 100 kHz–300 GHz fields, leads to the conclusion that field exposure below the thermal threshold is unlikely to be associated with adverse health effects (https://www.icnirp.org/en/frequencies/high-frequency/index.html accessed August 2018). This also means that exposure to high levels of EMFs can trigger heat-related biological effects, and therefore exposure levels are currently regulated by different guidelines (http://www.who.int/peh-emf/standards/en/). Nevertheless, according to the World Health Organization (WHO), “the current debate is centered on whether long-term low level exposure can evoke biological responses” (http://www.who.int/peh-emf/about/WhatisEMF/en/index1.html accessed August 2018).

In order to simulate the exposure to radiofrequency fields, which are present near radio frequency sources, such as the ones used by the members of the armed forces, we have previously developed different radiofrequency applicators^5,13^. The latter can be coupled to a light microscope and enable the real-time visualization of potential biological effects occurring during samples exposure to electromagnetic signals. In our previous studies, we have thus evaluated the effects of high power electromagnetic (HPEM) waves on GUVs and living cells^14^. The GUVs were chosen because they represent the simplest membrane model. Any alterations in their size would indicate a potential effect of electromagnetic waves on the phospholipid bilayer. Such assumption relies on the fact that when GUVs are submitted to pulsed electric field, which affects the integrity of the bilayer, the vesicles undergo fusion or fission^15^. While pulsed electric field affected GUV’s membranes, the exposure to HPEM waves did not induce any variations in size^14^.

In this study we addressed a number of biological effects that are generally observed when cells (including cellular spheroids) are submitted to permeabilizing pulsed electric field. Pulsed electric field may induce a series of effects^16^, including morphological and growth alterations of spheroids, which are tightly related to the loss of cell membrane integrity and ATP depletion^17^. In a first approach, which tried to assess the biological effects of the exposure of multicellular spheroids to HPEM waves, we have submitted cellular spheroids to radiofrequency fields, generated in field experiments by high power antennas, delivering moderate band (or meso-band) and narrowband signals. Under our exposure conditions the applied signals neither altered spheroids morphology nor did they alter spheroids growth. In the second approach, we have submitted spheroids to HPEM generated within dedicated applicators, which allowed real-time monitoring of the specimen with a microscope, during the illumination protocol, and allowed retrieving the samples, in order to submit them to further biological tests. Within the applicators, no effect was noted in terms of spheroids aspect, growth, plasma membrane integrity, induction of apoptosis, ATP content, and mitochondrial potential. We have decided to focus on the mentioned parameters because they are generally altered when the biological samples are exposed to permeabilizing electric fields. Normal and tumor cells were chosen because of their different growth rate, and because healthy and tumor cells might have a different proliferation rate when exposed to the electromagnetic field^18^. Nevertheless, under our exposure conditions, and for the parameters tested, no effects could be detected after spheroids exposure to applied signals.

Indeed, while thermal effects probably do not occur after moderate band 150 MHz HPEM signal exposure, potential biological effects might occur due to EMW-induced heating, occurring during narrowband 1.5 GHz signal exposure. Under our exposure conditions, a significant temperature increase (12.6 °C) was measured in field experiments. At the laboratory scale, the temperature increase was much lower due to conduction cooling, and attained 2.6 °C under harshest conditions (100 000 pulses, 500 Hz, 20 kV/m). At laboratory scale, the liquid medium heated the ceramic container, which subsequently transmitted its heat to the metallic parts of the applicator, while in field experiments there was less cooling by conduction.

## Methods

### Cell culture

Human tumor cell line HCT-116 was purchased from ATCC (catalog number #CCL-247). Human normal primary dermal fibroblasts were isolated from a skin biopsy provided by Icelltis (Toulouse, France) as previously described^19^. Both tumor and normal cells were cultivated in DMEM culture medium (Gibco, Invitrogen) supplemented with 10% fetal bovine serum (Gibco, Invitrogen) and 100 U/mL penicillin and 100 μg/mL streptomycin. Cells were cultured at 37 °C in a humidified atmosphere containing 5% CO_2_. Both cell types were regularly tested negative for mycoplasma using MycoAlert mycoplasma detection kit (Lonza #LT07-318).

### Spheroid formation

Spheroids were produced by cell seeding into an ultra-low attachment environment as previously described^17,20,21^. Briefly, 5000 cells in suspension were seeded in ultra-low attachment 96-well plates (Corning #7007) in 300 µL of cell culture medium. In summary, a volume of 30 mL of cDMEM medium containing 500 000 HCT-116 cells or normal dermal fibroblasts was prepared and placed in a disposable multichannel pipette reservoir, from which 300 µL aliquots were pipetted with a multichannel pipette and transferred into ultra-low attachment 96-well plates. This method allowed the obtention of spheroids with a very small variation in size, which equals in the case of HCT-116 cells to a spheroid volume variation of +/−1% at D2 after cell plating and +/−3% at D9, while for fibroblasts the size variation is +/−4% of the volume at D2 after cell plating to +/−5% at D9. The spheroids were randomly divided in different groups prior the treatment. Five days after seeding, the spheroids were ready for manipulation. Normal fibroblast spheroids were smaller that tumor spheroids as normal cells have a smaller proliferation rate and exhibit contact inhibition.

### Cellular spheroids exposure to HPEM signals

#### Signals generated in field experiments by high power antennas

**Figure 8:**
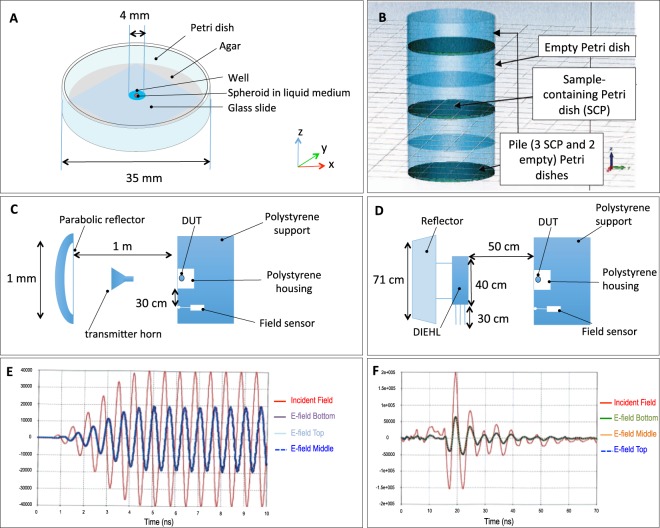
Field experiments experimental settings and electromagnetic signals. (**A**) The Petri dish containing the sample. The bottom of the dish is covered with 2% agar gel resulting in a 1 mm thick layer, containing a well in the middle, where the cell spheroid is placed. The well is covered with a glass slide. (**B**) A pile of Petri dishes, as used for simultaneous exposures. (**C**) Scheme of the narrowband signal exposure set-up. (**D**) Scheme of the moderate band exposure set-up. (**E**) Comparison of the electric fields after narrowband exposure, where the incident wave is labeled in red, the field within the top Petri dish is in light blue, in the middle Petri dish in dark blue and in the bottom Petri dish in purple color. The E fields within the samples have the amplitude of about 48.5% of the incident wave. (**F**) Comparison of the electric fields after moderate band exposure, where the incident wave is labeled in red, the field within the top Petri dish is in blue, in the middle Petri dish in orange and in the bottom Petri dish in green color. The E fields within the samples have the amplitude of about 31.5% of the incident wave.

Electromagnetic signals were generated by the narrowband electromagnetic signals were generated by the TEMPETE high power system (GERAC Electromagnetisme, Gramat, France) and the meso-band system DIEHL DS 110 (Diehl BGT Defence, Überlingen, Germany) (Fig. 1A–G). In the case of exposure of Petri dishes (Fig. 8A,B) in field experiments, the incident fields, delivered by antennas, were measured *in situ*, using an electric field sensor (in case of narrowband signals we used the Prodyn ACD-2 sensor and in case of meso-band signals we used the sensor EGG ACD7C, both sensors were coupled with balun EG&G DMB-4) and an oscilloscope (Tektronix TDS7404). The sensors were placed, in a first instance, at the location of the Petri Dishes, and subsequently, during the experimentations, at 30 cm from the Petri dish (Fig. 8C,D), in order not to distort the electric field around the Petri dish. Moreover an amplitude correction factor was applied, in order to take into account the attenuation, which depends on the relative positions of the antenna, of the Petri dish and of the final position of the E field sensor; thus we deduced the amplitude of the incident field at the position of the Petri dish. The amplitude of the coupled E-field into the solution was then deduced by applying a coupling coefficient, as previously computed using 3D simulations, to the estimated incident field; this coefficient is orientation dependent (H, E or k orientations) as discussed in previously^6^. During our experimentations, we used the H orientation (H vector in the same direction than the longitudinal axis of the Petri dish). The signals were acquired with fast digital oscilloscopes (Tektronix TDS7404).

Prior the illuminations in field experiments, the spheroids were placed in Petri dishes, which were conditioned with a 1 mm thin layer of agar gel (prepared with 2% Agar (A1296, Sigma Aldrich) in water, which contained a well (2r = 4 mm) within the gel. This well served as a reservoir where we placed the liquid, containing the spheroid (Fig. 8A). This volume extension with agar gel was used to homogenize the electric field within the sample, and the electric parameters at 1.5 GHz were the following: σ(solution) = 1.73 S/m, εr(solution) = 73.75; σ(Agar 2%) = 0.37 S/m, εr(Agar 2%) = 76. while at 200 MHz, we considered σ(solution) = 0.783 S/m, εr(solution) = 78; σ(Agar 2%) = 0.05 S/m, εr(Agar 2%) = 76. These parameters were considered as constant on the signal band, and were obtained with a dielectric probe (Hewlett-Packard 850708) and a network analyzer (Agilent PNA-L N5230C). The conductivity was calculated from the measures of the “epsilon second”: σ = (ε″.F)/18.109.

The experimental setting was organized as follows. The Petri dish (Fig. 8A (or a pile of 3 Petri dishes intercalated with two empty Petri dishes (Fig. 8B) – altogether referred to as device under test (DUT), were placed in front of the source as depicted in Fig. 8C,D. The DUT was placed in a polystyrene box, which is transparent to electromagnetic signals and was placed on a polystyrene support, placed 1.5 m above the ground and 1 m away from the center of the emission axis of the antenna. An electric field sensor was placed in the vicinity of the device under test-DUT (the spheroid-containing box) at an equal distance from the antenna as the DUT, either in the same axis, or at a position that was laterally shifted for 30 cm. The electric field within the solution inside one Petri dish (not shown in figure) or within each solution within the pile of three petri dishes (Fig. 8E,F) was almost identical.

The spheroids underwent either a single electromagnetic waves exposure protocol or a double exposure, performed in a 24 h interval. Non-illuminated spheroids (which were put in the same field but were placed in a Faraday cage) were used as control. Applied parameters are summarized in Fig. 1G.

#### Numerical evaluation of the electric field in the DUT

The electric field within the sample was evaluated by numerical simulations made with the CST Microwave studio software (the simulation parameters included: a time domain solver, the frequency of 1–2 GHz or 50–500 MHz for narrowband or moderate band signal simulations, respectively, the source in the plane wave, wave propagation in y, polarization in x, the excitation signal as actually measured within the system, Open Add Space boundary). In case of the narrowband simulation, taking into account the formula Dist = 2D^2^/λ, where “D” is the greatest dimension of the antenna, at the frequency of 1.5 GHz and a parabola of 1 m of diameter, numerical simulations indicated that the plane wave condition could be obtained if the DUT would be positioned 10 m from the source. This condition would not allow us to attain the desired electric fields. In order to simplify the numerical simulation, we considered the amplitude of the plane wave being equal to the one measured in the in the near field. As the measured signal was too long, we truncated it to shorten the time of the calculations. Conversely, in the case of moderate band signal simulations, while our experimental conditions did not fully satisfy the far field (plane wave) condition, we used the plane wave excitation type during the numerical simulation. Indeed, the Dist = 2D^2^/λ, at the dominant frequency of 200 MHz and a dipole of 70 cm of length, provides the far field at 63 cm. Nevertheless, as this distance would not allow us to attain desired electric field levels, the DUT was placed 50 cm from the source.

### Thermal effects

Potential thermal effects were evaluated with the thermal solver CST simulation software. The simulation was obtained using the same parameters as in electric field simulations.

#### Narrowband (TEMPETE) signals

The CST calculated the dissipated power density (at 1.5 GHz at 40 kV/m) of 1.287 × 10^9^ W/m^3^, which equals 1.287 × 10^3^ J/g/s for a sample density equaling the density of the water (10^6^ g/m^3^. If we neglect the cooling of the sample, the heating expressed in °C/s can be obtained by dividing 1.287 × 10^3^ J/g/s by the thermal capacity of the medium. Considering that the thermal capacity is equivalent to the thermal capacity of the water (4.18 J/g/K), the ΔT/dt = 1.287 × 10^3^ Jg^−1^ s^−1^/4.18 JK^−1^ g^−1^, resulting in ΔT/dt = 3.079 × 10^2^ °Cs^−1^ (as ΔT in °C = ΔT in K). For a signal excitation time of 4 μs, the ΔT = 3.079 × 10^2^ °Cs^−1^ × 4 × 10^−6^ = 1.23 × 10^−3^ °C per pulse, and we thus obtain a theoretical value of 6.158 °C after the application of 5000 pulses, if we neglect heat dissipation.

In practice, experimental measurements, performed at an ambient temperature of 8 °C and obtained after inserting a fiber optic thermometer (Nomad Fiber Optic Thermometer Model NMD 535 A coupled to the T1C-02-B05-DCU sensor and a 15 m EXT-3MP-15-DCU optic fiber) in a Petri dish (devoided of the cover), containing the agar extension and the liquid solution (as depicted in Fig. 8A), placed in the polystyrene box facing the antenna, and exposed to a target peak value of 40 kV/m, measured incident peak value 42 kV/m, width at half maximum 4 µs, computed (using 3D simulations) coupling coefficient 0.417, estimated internal field 17.48 kV/m, PRF 500 Hz, 5000 pulses per burst, 20 bursts and time between bursts 15 s, we detected a temperature increase of 12,6 °C.

If thermal effects would have to be avoided, the number of pulses per burst should be decreased or the delay between bursts should be increased.

#### Moderate band system (DIEHL) signals

The CST provided the temporal variation of power density. Taking into account the peak value attained in the DUT, and considering a sinusoidal signal, the value of the of power density is given by the following equation:

Power Density _RMS_ = 7.66 × 10^9^ Wm^−3^/√2 = 5.42 × 10^9^ Wm^−3^. In case of the sample density of 10^6^ g/m^3^, we thus obtain 5.42 × 10^3^ J/g/s.

If we neglect the cooling of the sample, the heating can be obtained by dividing the mentioned value by the thermal capacity of the medium. Considering that the thermal capacity is equivalent to the thermal capacity of the water (4.18 J/g/K), the ΔT/dt = 1296 °Cs^−1^. Under our experimental conditions, the signal had a damped amplitude (Fig. 1E), nevertheless, if we would not consider a damped signal, the temperature increment per pulse would equal ΔT = 1296 °Cs^−1^ × 2 × 10^−8^ s = 2.59 × 10^−5^ °C. Therefore, if there would be no heat dissipation, we theoretically obtain a temperature increase of 0.0647 °C after 2500 pulses.

In practice, experimental measurements, performed at ambient T = 8 °C, with the materials/methods as described for narrowband signals, could not detect any temperature increase in the sample.

### Signals generated in laboratory experiments within dedicated applicators

In the case of exposure at the laboratory scale using transmission line applicators as described by Chrétiennot *et al*.^5^, the sample under test (=cellular spheroid in the culturing liquid) was placed within a high permittivity specific ceramic container in contact with the applicator’s electrodes (the transmission-line cell was of the transverse electromagnetic cell type). In this case, the E field, to which the sample is submitted, is directly obtained by the ratio E(V/m) = V(V)/d(m), where V equals the tension, measured at the output line, and d is the distance between the electrodes. The tension was measured at the output line through a 50 Ohm calibrated power attenuator (BARTH device). The signals were acquired with digital oscilloscopes.

The design of narrow- and meso-band applicators for spheroids illumination was described previously^5^, and enables optimal electric field coupling and optimized electric field homogeneity within the tested sample. The dimensions of the exposure devices have to be as small as possible, with regards to the size of the biological sample under test, to minimize the required voltage (limited to a few kV) delivered by the associated generators and to suppress or control the parasitic electromagnetic signals. Figure 3 shows the macroscopic views, schematic views and typical signals delivered by the narrowband (A) and meso-band applicator (B). The applicators are both stripline-based and the biological samples are placed into high permittivity ceramic bloc containers, which are in contact with the stripline conductors. As there are no air gaps between the sample and the conductors, a maximized coupling coefficient and an excellent E-field homogeneity are obtained^5^. For high frequencies, the half wavelength of the ceramic container constitutes a transmission line impedance transformer, allowing matching the 50 Ohm microstrip to a specific frequency (1.5 GHz). The main advantage of these applicators are: (1) the confinement of the systems, preventing the occurrence of radiations in the surrounding external environment, (2) the possibility to power the applicators with selected generators and amplifiers, which allows tuning several independent parameters (such as electric field strength, PRF, number of pulses, *etc*.), and (3) the possibility to retrieve radio-wave-exposed biological samples, allowing the samples to be re-cultured after exposure to electromagnetic waves.

The electromagnetic parameters applied during narrowband (NB) illumination and meso-band (MB) illumination procedures are described in Fig. 3C,D, respectively. The illuminations were applied after placing the spheroids, suspended in 20 µL of cell culture medium, into the ceramic block within the applicator. The illuminated samples consequently underwent biological analyses.

The positive control consisted of spheroids submitted to the permeabilizing electric field, which is characterized by the field strength of 1000 V/cm, pulse duration of 100 µs, 8 pulses, applied at a frequency of 1 Hz^17^.

#### Thermal measurements performed at laboratory scale

In order to perform the thermal measurements, a hole was drilled into the lateral side of the applicator and the fiber optic thermometer (Nomad Fiber Optic Thermometer Model NMD 535 A coupled to the T1C-02-B05-DCU sensor and a 15 m EXT-3MP-15-DCU optic fiber) was inserted into the well of the ceramic container of the applicator, which contained the cell culturing medium. The temperature of the medium raised from 23.4 °C to 25 °C (after application of 20 bursts of 5000 pulses, delay between bursts 15 s, PRF 500 Hz, E-field strength 16 kV/m) and from 24.2 °C to 26.8 °C (after application of 2 bursts of 50 000 pulses, delay between bursts 15 s, PRF 500 Hz, E-field strength 20 kV/m). The application of 50 000 pulses, with delay between bursts 1 s, PRF 100 Hz, and E-field strength 20 kV/m led to the medium temperature increase of 0.6 °C. The E-field strength within the applicator had the approximate value of the internal E-field measured in field experiments (17.48 kV/m) after applying the radiated target peak value 40 kV/m.

#### Spheroids macroscopic aspect and growth

The macroscopic aspect and growth of spheroids were monitored prior the exposure to HPEM signal, immediately after illumination and during a period of 9 days after signal exposure. The imaging was made on entire (that is unsliced) spheroids, imaged in the cell-culturing medium. The morphology and surface were followed by wide field light microscopy using a Leica DM IRB microscope (Leica Microsystems, Wetzlar, Germany) coupled to a CoolSNAP HQ camera (Roper Scientific, Photometrics, Tucson, USA). The imaging was made after setting the focus on the equatorial plane, which gave the largest diameter of the observed spheroid. Spheroids exhibited spherical, ellipsoid, or ovoid shapes. The equatorial plane was used to determine the area of reference (expressed in µm²), which was then used for the assessment of spheroids growth. Spheroid size (area in µm²) was determined using Image J software (NIH, Bethesda, USA) as previously described^17,20^.

#### Plasma membrane integrity assessment in illuminated cellular spheroids

Plasma membrane integrity was evaluated with a small fluorescent probe, the propidium iodide (Sigma-Aldrich #P4170). Propidium iodide is a non-permeant DNA intercalant, which becomes highly fluorescent when intercalated in DNA of cells presenting plasma membrane defects. Propidium iodide can thus reveal the integrity of cells covering spheroids surface and the cells in deeper layers of the spheroids, namely within the necrotic core^22^. Briefly, immediately after illumination, spheroids were incubated at room temperature for 5 min in 100 µM propidium iodide diluted in PBS. Red fluorescence was detected with a wide field fluorescence microscope (Leica DM IRB microscope) coupled to a CoolSNAP HQ camera^17,20^.

#### Proliferation detection in illuminated cellular spheroids

Cell proliferation was detected with a commercially available kit (Click-iT™ EdU Cell Proliferation Kit for Imaging, Alexa Fluor™ 488 dye, #C10337) according to the manufacturer’s protocol. Briefly, 24 h after illumination, the spheroids were incubated in the reactive solution containing specific substrates. The Alexa Fluor® dye containing EdU (5-ethynyl-2′-deoxyuridine), which is provided in the kit, is a nucleoside analog of thymidine and is incorporated into DNA during active DNA synthesis, evidencing the proliferating cells. Blue fluorescent Hoechst 33342 dye, provided in the kit, was used for cell cycle analysis. Labeled spheroids were imaged with a wide field fluorescence microscope (Leica DM IRB microscope), coupled to a CoolSNAP HQ camera (Roper Scientific, Photometrics).

#### Apoptosis detection in illuminated cellular spheroids

Apoptosis was detected using a commercially available kit (Image-iT Live Red Caspase-3 and -7 detection kit for microscopy, #I35102, Molecular probes, Invitrogen, France) according to the manufacturer’s protocol. Briefly, 1 h after illumination the spheroids were incubated in the reactive solution containing specific substrate for caspases-3/7. When activated, these caspases cleave their substrate, releasing a fluorescent probe, which can be detected with a wide field fluorescence microscope (Leica DM IRB microscope), coupled to a CoolSNAP HQ camera (Roper Scientific, Photometrics). Thus, the red-fluorescent signal is a direct measure of the amount of active caspases. In the positive control, apoptosis was chemically induced by 1 µM staurosporine (STS, Sigma), a known pro-apoptotic agent, in an incubation protocol lasting 24 h adapted from ref.^23^.

#### ATP content measurement in illuminated cellular spheroids

Spheroids ATP content was determined with Cell-Titer Glo 3D viability assay (#G9681, Promega, France), according to the manufacturer’s protocol. Briefly, 5 minutes after illumination, the spheroids were rinsed in a large volume of PBS. Eighty microliters of PBS containing one spheroid were then deposited per well in a white 96-well plate, and 80 μL of the reagent were immediately added to the wells. Spheroids were incubated under gentle rotation for 30 min at room temperature to allow tissue lysis. ATP content was measured by light emission using a luminometer (Clariostar, BMG Labtech, Germany).

#### Mitochondrial potential observation in illuminated cellular spheroids

In order to monitor mitochondrial membrane potential in cells, we used MitoView 633 (absorbance/emission at 622/648 nm) (#BTM70055, Ozyme, France). This dye is membrane permeable and becomes fluorescent upon accumulation in the mitochondria. Briefly, spheroids were incubated for 30 min at 37 °C in cell culture medium supplemented with MitoView 633 at 50 nM. Fluorescence signal was detected before illumination and 5 minutes after with a wide field fluorescence microscope (Leica DM IRB microscope) coupled to a CoolSNAP HQ camera (Roper Scientific, Photometrics).

### Statistical analysis

Data were obtained from 6 independent experiments and for each experiment 12 spheroids were analyzed. Data are expressed as mean ± SEM.

## Supplementary information

Supplementary information

## Data Availability

The data generated during and/or analyzed during the current study are available from the corresponding author on reasonable request.
