# Supplementary material for: Evaluations of Acute and Sub-Acute Biological Effects of Narrowband and Moderate-Band High Power Electromagnetic Waves on Cellular Spheroids
Source: Sci Rep. 2019 Oct 25;9:15324. doi: 10.1038/s41598-019-51686-9 (PMC6814744; doi:10.1038/s41598-019-51686-9)
Supplement: Supplementary file 1 — Supplementary information [file 41598_2019_51686_MOESM1_ESM.docx]

**SUPPORTING INFORMATION for**

**Evaluations of Acute and Sub-Acute Biological Effects of Narrowband and Moderate-Band High Power Electromagnetic Waves on Cellular Spheroids**

Laure Gibot^1¤^, Jelena Kolosnjaj-Tabi^1¤^, Elisabeth Bellard^1^, Thomas Chretiennot^2^, Quentin Saurin^2^, Alexandre Catrain^2^, Muriel Golzio^1^, René Vézinet^2^, Marie-Pierre Rols^1^*

^1^ Institut de Pharmacologie et de Biologie Structurale, Université de Toulouse, CNRS, UPS, Toulouse, France

1. CEA, DAM, GRAMAT, F-46500 Gramat, France

* corresponding author: Marie-Pierre Rols; [rols@ipbs.fr](mailto:rols@ipbs.fr); Institut de Pharmacologie et de Biologie Structurale, 205 Route de Narbonne, 31400 Toulouse, France.

**Applicators and signals applied in laboratory experiments**

Electromagnetic fields calculated with CST Microwave studio for geometries shown in Figures 3A and 3B.

1. Applicator for narrowband signals

This subcentimetric applicator is of the stripline type, and the Teflon bloc serves as mechanical support, a hot conductor, and a support for the containers of the solution under test. The solution under test is placed in cylindrical containers (with a diameter of 3 mm and depth of 2 mm) made of high permittivity ceramics. The external dimensions of the ceramic parts were h= 4 mm and l = 15.4 mm. Such a length allowed the propagation of the wave within the ceramics (in order to create a section of length of λ/2 at 1.5 GHz for ξ=78). The schematic view is shown in figure S1.


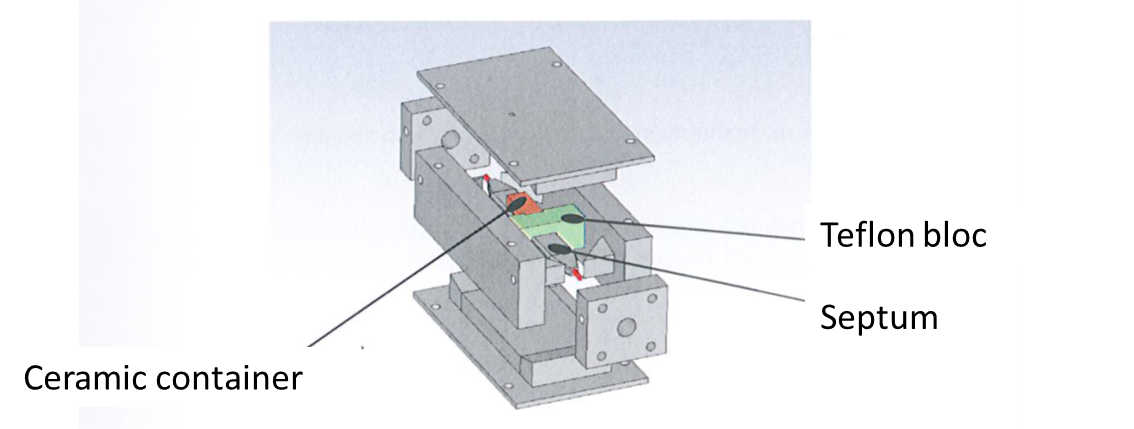


Figure S1. Schematic representation of the subcentimetric applicator for 1.5 GHz narrowband signals.

Electrical properties simulations

The simulations were made without taking into account the ceramic containers and with ceramic containers filled with a sample of deionized water (Figure S2). The following dielectric properties of the ceramic material were used:

ξ_R_ = 78,

tan(δ) = 1.10^-3^ at 1.5 GHz, and the dielectric properties of water (deionized) were the ones, which are available in the CST database.


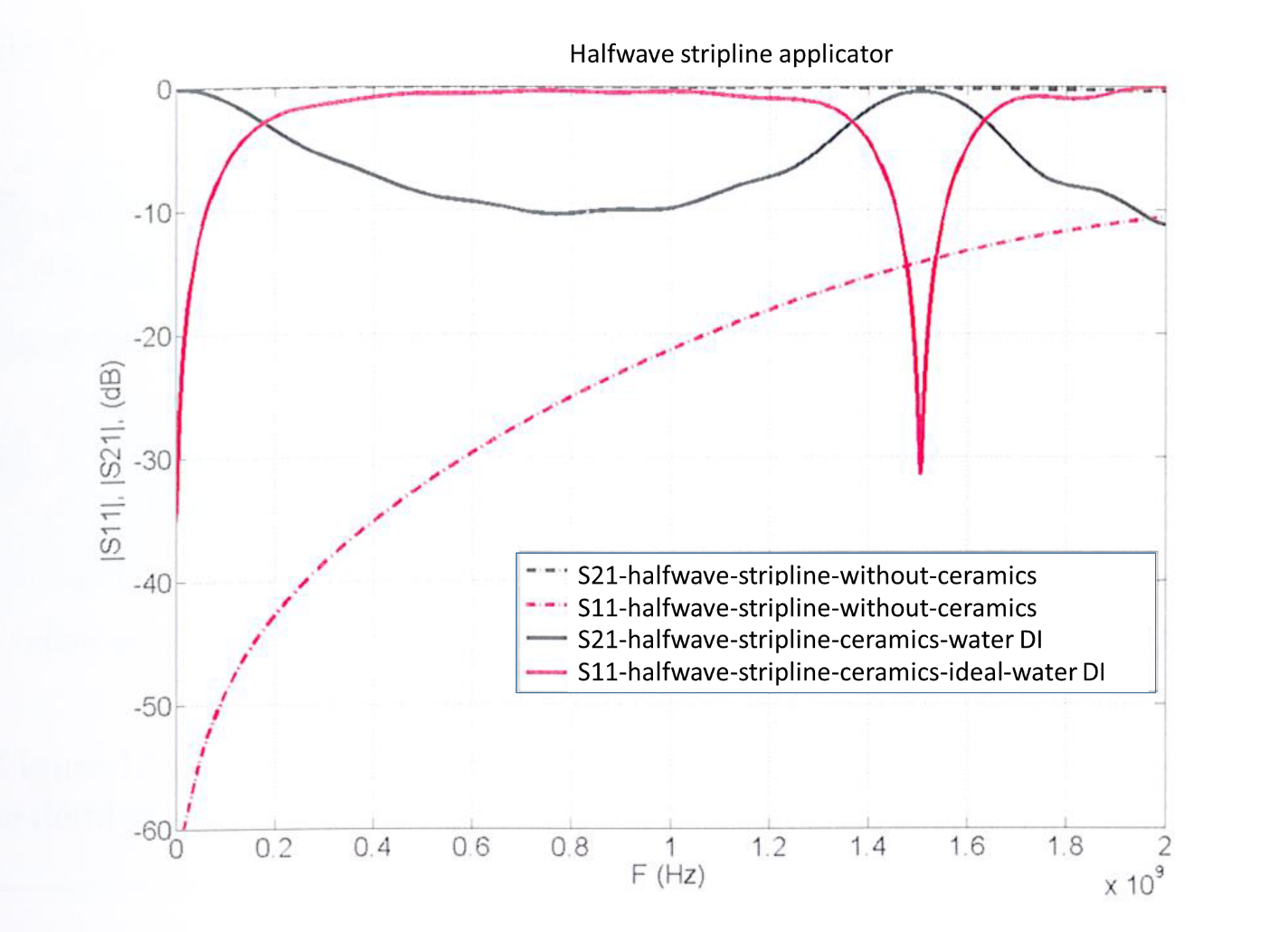


Figure S2. S11 and S21 parameters calculated for the λ/2 stripline-based applicator in absence and presence of the ceramic container filled with DI water.

1. Applicator for meso-band signals

This subcentimetric applicator is characterized by two containers made of high permittivity ceramics where the DUT is placed. The containers (dimensions 5x4x3 mm^3^ have a cylindrical hole (3 mm diameter and 2 mm depth) have the capacity of 14 µL. the schematic view is shown in figure S3.


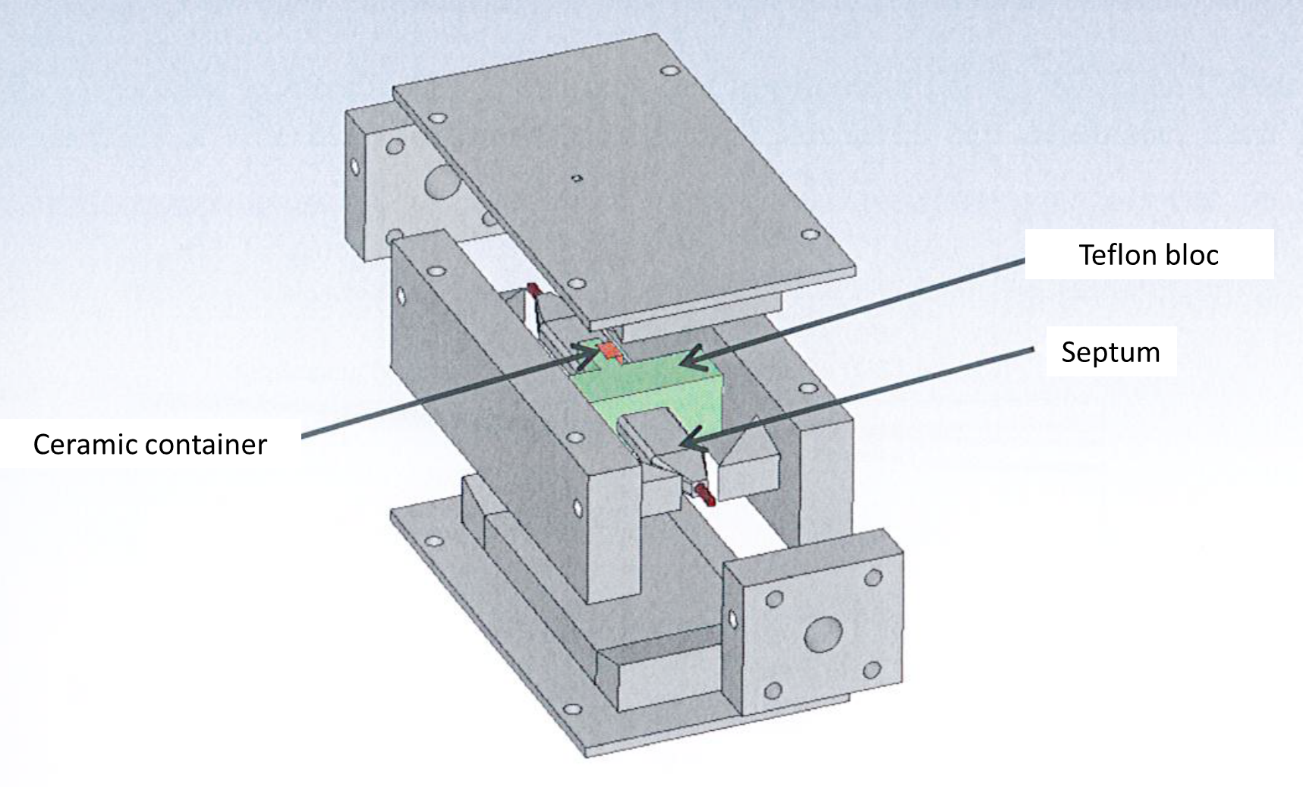


Figure S3. Schematic representation of the subcentimetric applicator for moderate band signals.

Electrical properties simulations

The same properties were used as for the λ/2 stripline-based applicator, namely ξ_R_ = 78,

tan(δ) = 1.10^-3^, at 200 MHz, while the dielectric properties of water (deionized) were taken from the CST database.


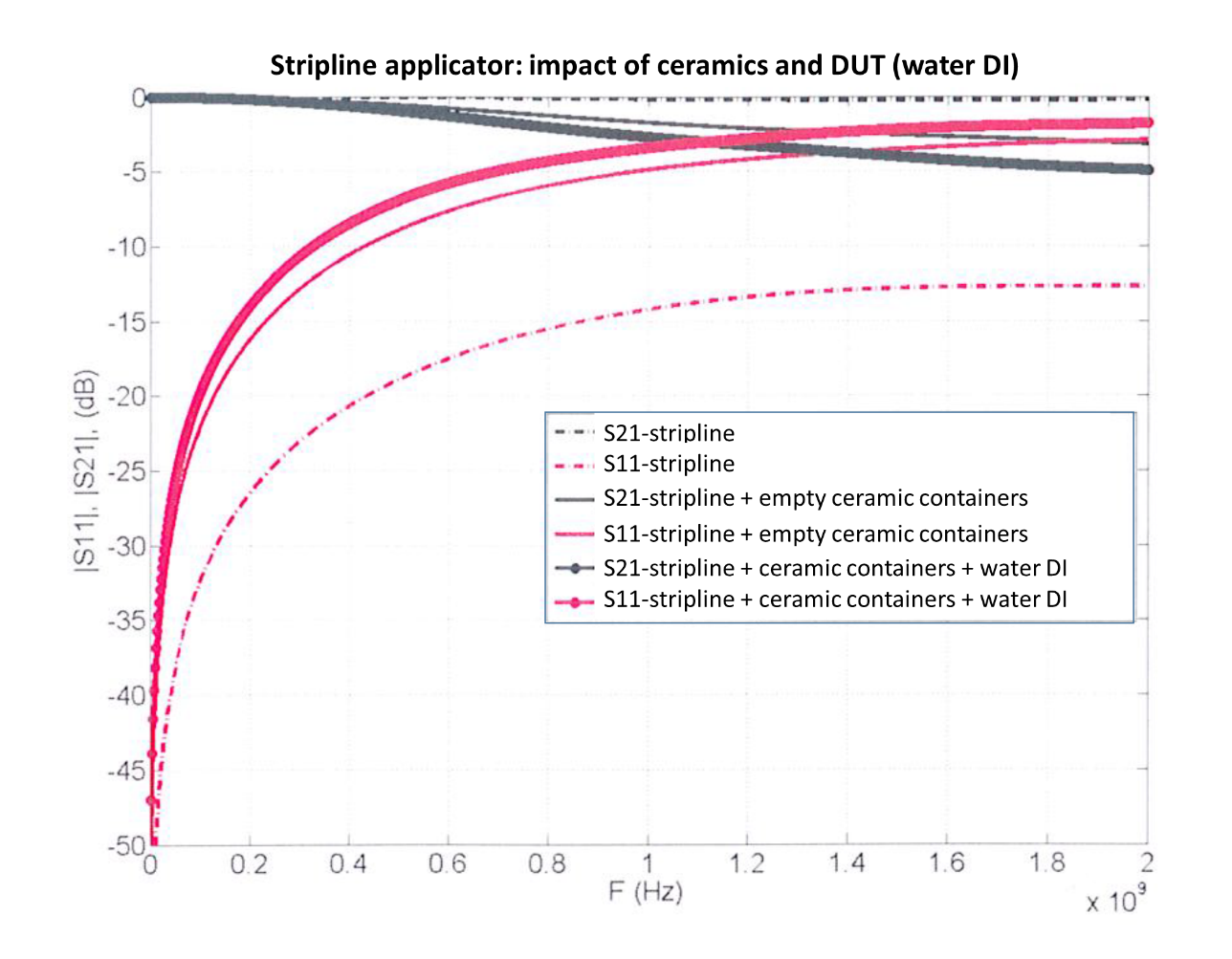


Figure S4. S11 and S21 parameters calculated for the applicator in absence and presence of the ceramic container filled with DI water.

**3D electromagnetic simulations with CST Microwave Studio EM Simulation Tool**

The homogeneity of the electric field was evaluated for both types of applicators (Figures S5 and S6) in two planes: 1) in the middle of the device under test (DUT), orthogonal to the direction of propagation and 2) parallel to the direction of propagation, going through the center of the hot conductor.


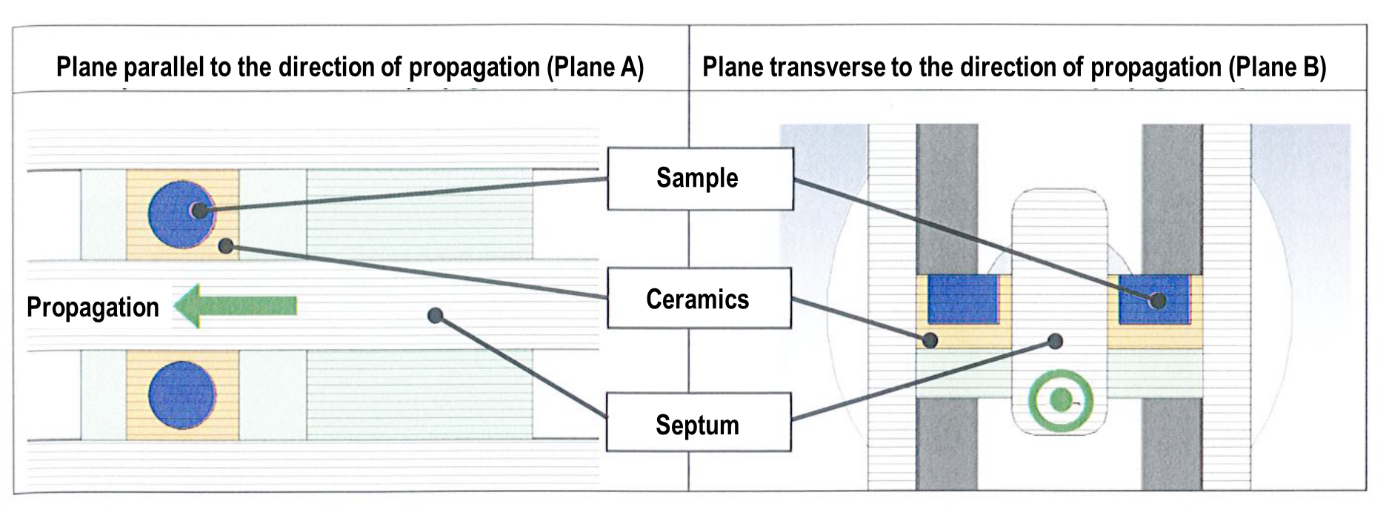


Figure S5. Cross sections used for computed evaluation of E-field homogeneity in the DUT in the meso-band applicator.


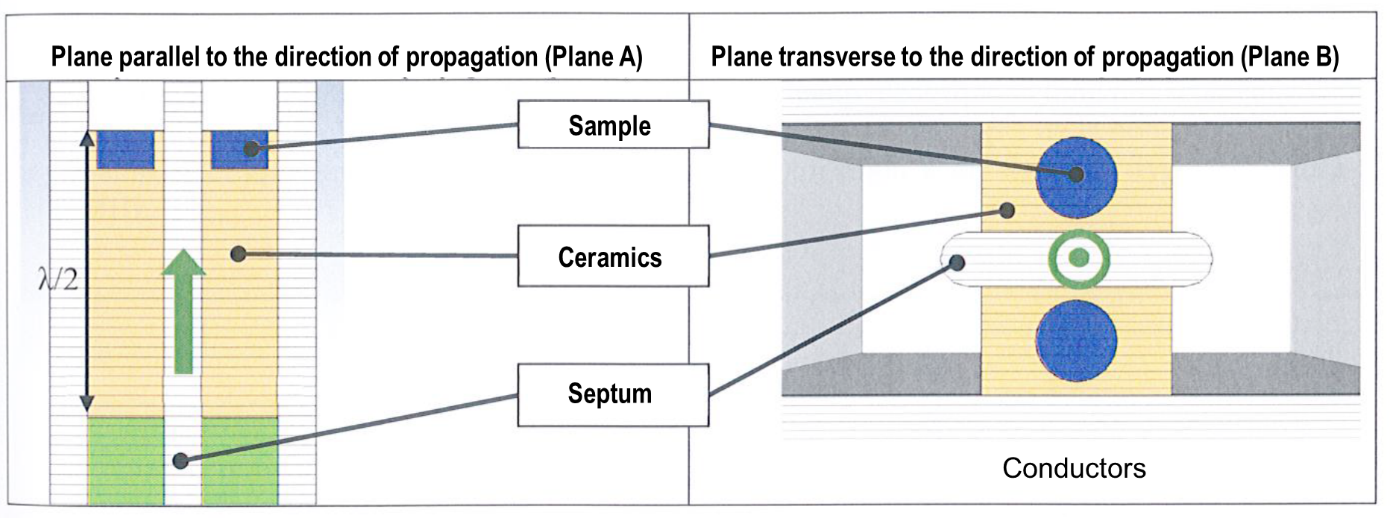


Figure S6. Cross sections used for computed evaluation of E-field homogeneity in the DUT in the λ/2 stripline-based applicator (applicator for 1.5 GHz narrowband signals).

The maxima of the electric field in the DUT in planes A and B are shown in figure S7 for the meso-band applicator and in figure S8 for the λ/2 stripline-based applicator.


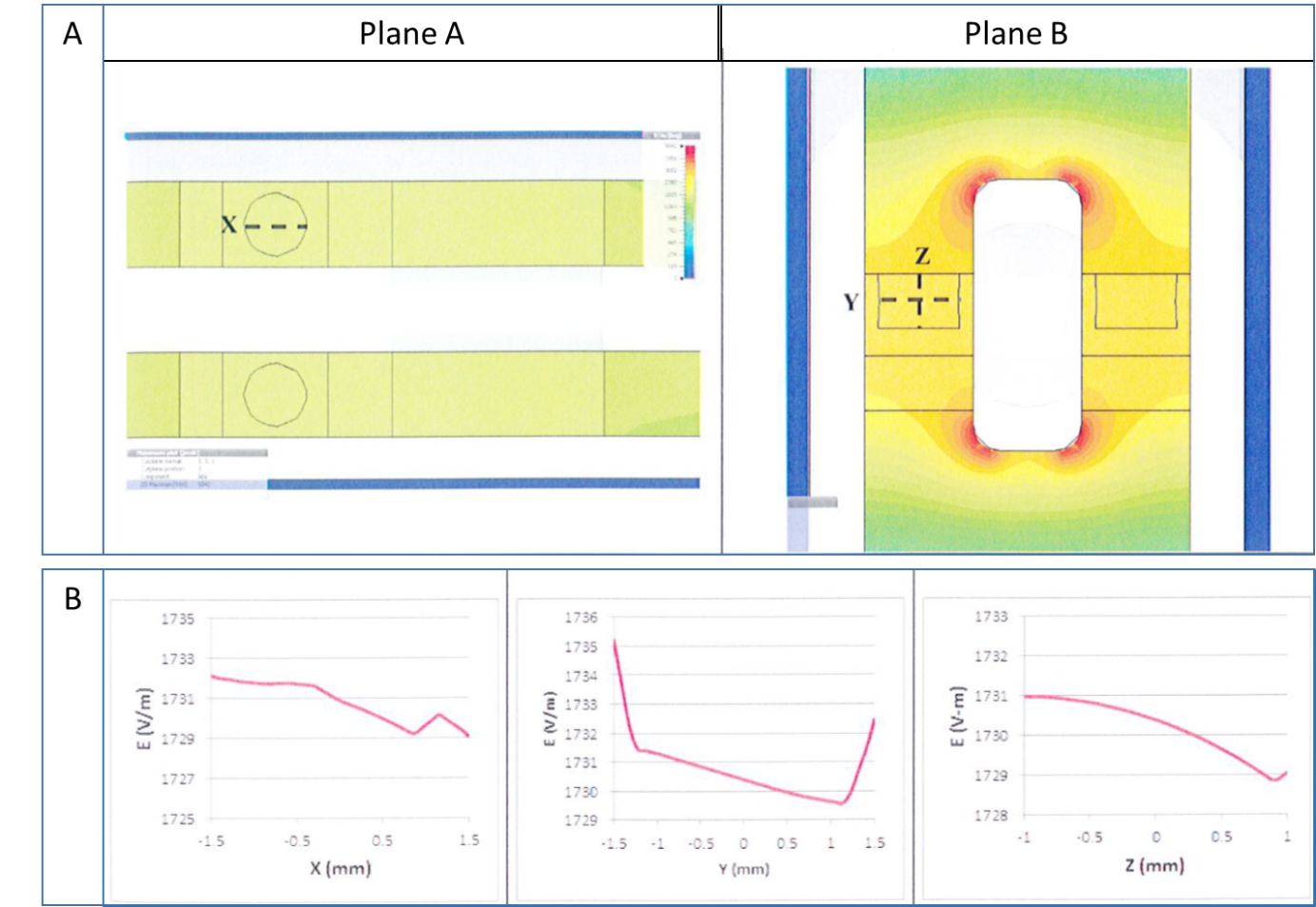


Figure S7. A) Electric field maximum in the DUT (planes A and B), for a meso-band signal centered at 200 MHz. B) Maximum amplitude of the electric field along X, Y and Z segments as shown in dashed lines on figure A on planes A and B.

The E-field homogeneity along segments X, Y and Z is given by the ratio

We thus obtain:

(1732-1730)/1732 < 1% for segment X,

(1735-1730)/1735 < 1% for segment Y,

(1731-1728)/1731 < 1% for segment Z, meaning that the E-field homogeneity is reached within 1%.


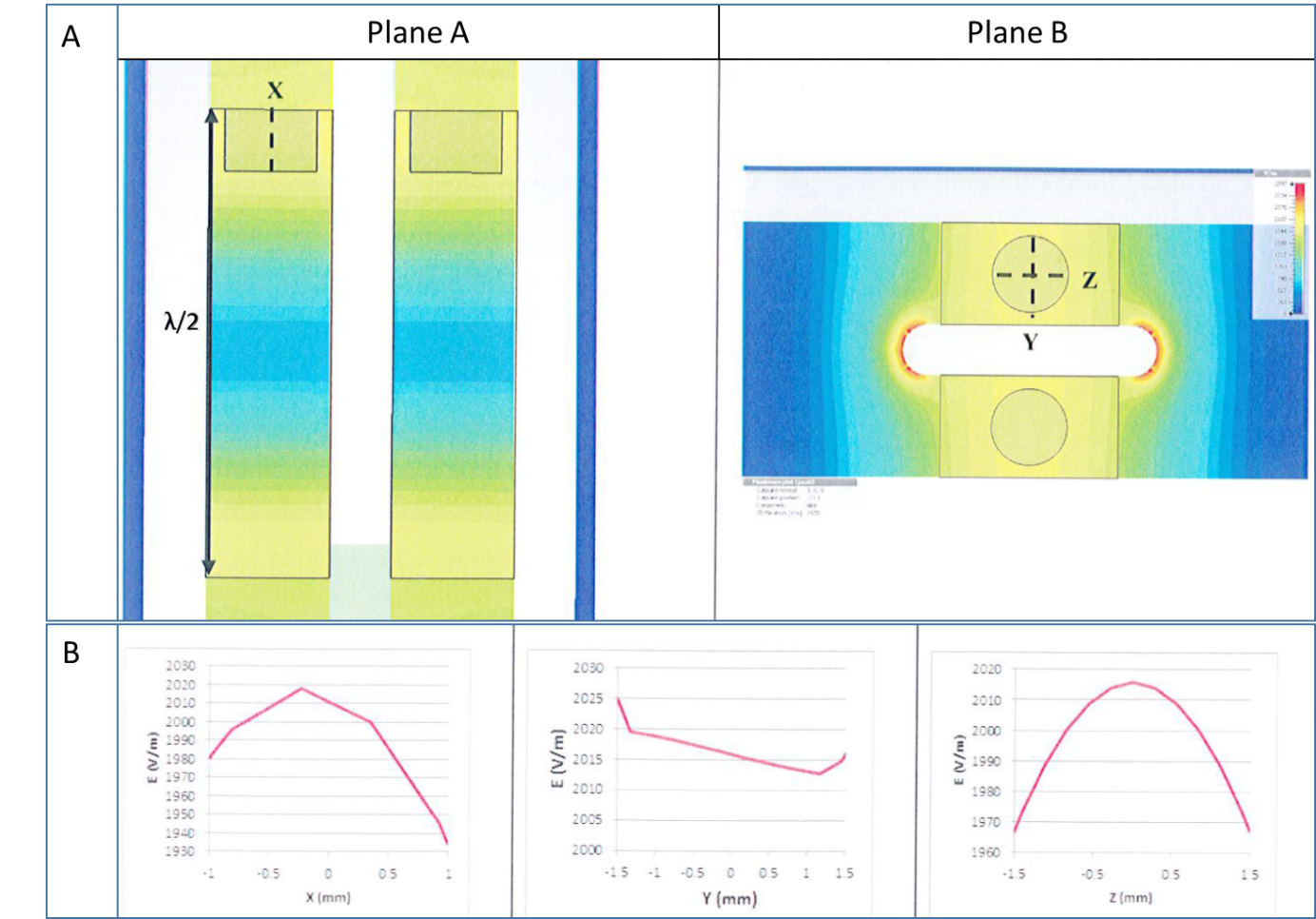


Figure S8. A) Electric field maximum in the DUT (planes A and B), for a narrowband signal centered at 1.5 GHz. B) Maximum amplitude of the electric field along X, Y and Z segments as shown in dashed lines on figure A on planes A and B.

We thus obtain:

(2020-1930)/2020 ≈ 4.5 % for segment X,

(2025-2012)/2025 < 1% for segment Y,

(2015-1965)/2015 ≈ 2.5 % for segment Z, meaning that the E-field homogeneity is reached within approximately 5%.
